# Application of three different methods to determine the prevalence, the abundance and the environmental drivers of culturable *Vibrio cholerae* in fresh and brackish bathing waters

**DOI:** 10.1111/jam.13940

**Published:** 2018-08-13

**Authors:** A.K.T. Kirschner, S. Pleininger, S. Jakwerth, S. Rehak, A.H. Farnleitner, S. Huhulescu, A. Indra

**Affiliations:** ^1^ Institute for Hygiene and Applied Immunology Water Hygiene Medical University Vienna Vienna Austria; ^2^ Interuniversity Cooperation Centre for Water & Health Vienna Austria; ^3^ Institute for Medical Microbiology and Hygiene Austrian Agency for Health and Food Safety Vienna Austria; ^4^ Institute of Chemical, Environmental & Bioscience Engineering Technische Universität Wien Vienna Austria; ^5^ Research Department Water Quality and Health Karl Landsteiner University of Health Sciences Krems Austria

**Keywords:** conductivity, cultivation, direct plating, infection, membrane filtration, most probable number, *Vibrio cholerae* nonO1/nonO139

## Abstract

**Aims:**

Three cultivation methods were used to study the prevalence and abundance of *Vibrio cholerae* in Eastern Austrian bathing waters and to elucidate the main factors controlling their distribution.

**Methods and Results:**

*Vibrio cholerae* abundance was monitored at 36 inland bathing sites with membrane filtration (MF), a standard most probable number (MPN) approach and direct plating (DP). Membrane filtration yielded the most reliable and sensitive results and allowed *V. cholerae* detection at 22 sites with concentrations up to 39 000 CFU per 100 ml, all belonging to serogroups other than O1 and O139 and not coding for cholera toxin and toxin coregulated pilus. Direct plating turned out as an easy method for environments with high *V. cholerae* abundances, conductivity was the only significant predictor of *V. cholerae* abundance in the bathing waters at warm water temperatures.

**Conclusions:**

*Vibrio cholerae* nonO1/nonO139 are widely prevalent in Eastern Austrian bathing waters. Instead of the standard MPN approach, MF and DP are recommended for *V. cholerae* monitoring. Conductivity can be used as a first easy‐to‐measure parameter to identify potential bathing waters at risk.

**Significance and Impact of the Study:**

*Vibrio cholerae* nonO1/nonO139 infections associated with bathing activities are an increasing public health issue in many countries of the northern hemisphere. However, there are only limited data available on the prevalence and abundance of *V. cholerae* in coastal and inland bathing waters. For monitoring *V. cholerae* prevalence and abundance, reliable and simple quantification methods are needed. Moreover, prediction of *V. cholerae* abundance from environmental parameters would be a helpful tool for risk assessment. This study identified the best culture‐based quantification methods and a first quick surrogate parameter to attain these aims.

## Introduction

*Vibrio cholerae* is one of the most important waterborne bacterial pathogens and the causative agent of epidemic cholera. Only strains belonging to serogroups O1 and O139 and possessing the virulence factors cholera toxin (ctx) and toxin coreglated pilus (tcp) are able to cause this devastating disease. Besides cholera, nontoxigenic *V. cholerae* strains (belonging to serogroups other than O1 and O139) are able to cause a variety of other infections such as gastroenteritis, ear, wound, blood or soft‐tissue infections (Huhulescu *et al*. 2007; Crowe *et al*. 2016; Engel *et al*. 2016). In the past two decades, *Vibrio*‐associated infections have been significantly increasing in Europe and the United States of America (Newton *et al*. 2012; Baker‐Austin *et al*. 2013; Le Roux *et al*. 2015; Vezzulli *et al*. 2016). Especially in Northern Europe (Baltic Sea), infections of swimmers have been predominantly caused by *V. cholerae* nonO1/nonO139 (Baker‐Austin *et al*. 2016b). Some serious infections of swimmers due to *V. cholerae* nonO1/nonO139 have also been reported from other European countries (Stypulkowska‐Misiurewicz *et al*. 2006; Huhulescu *et al*. 2007; Sterk *et al*. 2015; Dobrovic *et al*. 2016; Engel *et al*. 2016; Hirk *et al*. 2016; Maraki *et al*. 2016). The observed increase in infection frequency has been linked to global warming and the significantly increased water temperatures observed in many aquatic environments (Baker‐Austin *et al*. 2016b; Vezzulli *et al*. 2016). Average water temperatures of the North Sea or the American North Atlantic Coast have increased by on average 1·5°C in the past 30 years (Vezzulli *et al*. 2016) and maximum values 10°C above the long‐term average have been reported during a recent heatwave in the Baltic Sea (Baker‐Austin *et al*. 2016b). It is well known that higher temperatures enhance growth of *V. cholerae* and other Vibrios (Huq *et al*. 1984; Kirschner *et al*. 2008), but whether the observed increases in infection cases are related to an increase in *Vibrio* concentrations or to higher exposure (a higher number of swimmers can be expected at higher water temperatures), still has to be proven. From the retrospective molecular biological analysis of plankton samples Vezzulli *et al*. (2012, 2016) demonstrated that the concentration of Vibrios attached to zooplankton has significantly increased in the past 30 years, linked to the significant increase in water temperatures in different marine environments. In Austria, two extremely severe cases of necrotizing fasciitis occurred for the first time in 2015. Both cases were associated with bathing activities in two ponds (Hirk *et al*. 2016) and occurred in August during an extreme summer heatwave within the second warmest year recorded in our country (http://www.zamg.ac.at). So far, endemic *V. cholerae* infections have been associated only with the brackish lake Neusiedler See (Huhulescu *et al*. 2007) known to harbour high concentrations of *V. cholerae* nonO1/nonO139, as is the case in other saline alkaline lakes nearby (Schauer *et al*. 2015).

Due to the dramatic cases of necrotizing fasciitis in 2015, in the following two years a program was initiated to monitor the prevalence and abundance of culturable *V. cholerae* in a wide range of ecologically different bathing waters in Eastern Austria. The sampled waters were selected along a gradient of electrical conductivity ranging from 290 to 2650 *μ*S cm^−1^ (corresponding to approx. salinities of 0·14–1·4 ppt) because salinity is a main predictor of *V. cholerae* growth (Singleton *et al*. 1982). In order to get reliable quantitative information, different methods were applied to quantify culturable *V. cholerae*. In addition to the current standard protocol based on a most probable number (MPN) approach with enrichment in alkaline peptone water (APW) (Public Health England 2005; ISO 2007; Huq *et al*. 2012; CDC 2014) we used a membrane filtration (MF) protocol that was already successfully applied in the lake Neusiedler See (Schauer *et al*. 2012, 2015) and a simple direct plating (DP) protocol as suggested by Huq *et al*. (2012). Concomitantly, basic environmental parameters were recorded in order to establish a prognostic model for prediction of *V. cholerae* concentrations at Eastern Austrian bathing sites.

## Materials and methods

### Sampling

Water samples were taken from June to October 2016 and in July 2017 from 36 different bathing sites in Eastern Austria. Austria is situated in the temperate climate zone and warm water temperatures allowing detection of culturable *V. cholerae* are restricted to the period between April and November with maximum *V. cholerae* concentrations observed between June and September (Schauer *et al*. 2015; Bliem *et al*. 2018). The sites were chosen by means of salinity and included a large brackish lake, several smaller brackish lakes and ponds, several small freshwater lakes and ponds as well as a few river bathing sites. In total, 89 samples were analysed with seven sites measured six times (sites 27–33), three sites measured five times (sites 20–21) and another three sites (sites 34–36) measured three times. The remaining 23 sites were measured once in July or August when water temperature conditions were high (21–27°C). Details on sampling dates at each bathing site can be found in Table S1. Four replicate samples were taken at each sampling event in one‐way sterile 500‐ml plastic bottles at the official EU bathing site locations or—in case of unofficial bathing ponds—at sites where the majority of people go bathing. All bottles were transported to the lab in cooling boxes at a temperature between 10 and 20°C and analysed within maximal 6 h after the first sample was taken. Three replicate bottles—each considered a separate sample—were used for the MF analysis, and one bottle was used for the MPN and DP assays. Processing of samples was conducted immediately after arrival in the lab at the Medical University of Vienna (MUW; MF) and the lab of the Austrian Agency of Health and Food Safety (AGES; MPN and DP). For the analysis, unfiltered, well‐mixed water samples were used, including both sediment particles and plankton. During sampling, a range of environmental parameters was recorded with portable meters: water temperature, electrical conductivity, pH value, oxygen content and water transparency (Secchi depth); an overview of the environmental data of the different sites can be found in Table 1.

**Table 1:** Overview of the measured basic ecological parameters of each investigated bathing site. For some sites multiple measurements over the season (*n* = 3–6) were performed. Values in brackets for the parameter ‘area of water body’ indicates that the respective bathing site is allocated to a defined small area, but belongs to a larger water body

Bathing site	Area of water body (km^2^)	Max. water depth (m)	Electrical conductivity (*μ*S cm^−1^)	pH value	Water temperature (°C)	Oxygen (% saturation)	Secchi depth (m)
1	1·97	6·3	322	8·65	21·0	113·1	>1·50
2	1·26	6·0	307	8·57	24·2	145·5	>1·50
3	0·27	2·5	416	8·28	23·1	107·5	>2·00
4	0·95	6	288	9·29	22·6	114·0	>2·00
5	0·16	3	332	8·26	23·5	118·4	>1·50
6	0·20	3	345	8·27	23·2	117·7	>1·50
7	0·03	2·5	431	8·12	22·2	117·4	>1·00
8	0·11	3·0	360	9·08	26·5	117·7	>2·00
9	0·61	6·8	640	8·00	24·0	102·7	>1·50
10	0·12	12	1385	8·44	22·8	93·9	>1·00
11	0·13	7·5	870	8·39	24·5	123·3	>1·00
12	0·04	10	900	8·64	24·9	121·5	>1·00
13	0·5	6	520	8·23	26·7	130·0	1·80
14	0·03	4	557	8·28	26·2	122·3	2·00
15	0·44	5·5	440	8·38	24·9	179·0	0·50
16	0·01	2·5	482	8·25	25·4	178·5	0·70
17	0·03	19	1721	8·59	26·5	122·6	3·00
18	0·02	11	1243	8·39	26·5	122·7	2·80
19	0·04	4	915	8·15	26·8	141·5	1·20
20	0·02	7	2240–2450	9·03–9·18	12·9–25·6	119·8–166·4	2·0–2·5
21	0·02	10	2390–2656	7·97–8·32	13·5–27·6	91·5–120	1·7–2·0
22	0·01	3	1258–1374	8·01–8·37	11·3–24·4	77·8–136	0·32–1·0
23	0·6	22	786	8·31	25·2	130·0	2·00
24	0·03	17	382	8·33	24·3	121·6	2·00
25	0·04	3	499	8·13	26·9	131·0	2·00
26	0·05	24	1736	8·30	26·0	124·2	1·60
27	0·04 (320)	1·8	1895–1988	8·64–9·08	19·5–25·2	95·8–101	0·12–0·42
28	0·04 (320)	1·8	1987–2140	8·51–8·93	19·8–25·9	83·7–96·2	0·23–0·32
29	0·03 (320)	1·8	2000–2070	8·38–8·89	19·6–26·2	83·7–95·6	0·18–0·31
30	0·03 (320)	1·8	1859–1995	8·45–8·94	19·6–26·1	77·7–104·1	0·17–0·37
31	0·04 (320)	1·8	1845–1970	8·51–9·02	20·6–26·5	91·0–101·8	0·18–0·35
32	0·02 (320)	1·8	1850–1976	8·59–9·03	10·7–26·7	87·4–104·4	0·20–0·35
33	0·22 (320)	1·8	1820–1973	8·61–9·11	20·4–26·4	100·1–106·3	0·15–0·43
34	0·03	6	1180–1200	8·24–8·68	23·9–27·0	118·2–130·9	1·8–2·5
35	0·02	6	1448–1460	8·53–8·93	23·7–27·1	117·3–129·2	1·7–1·95
36	1·8	1·4	1985–2020	8·26–8·85	21·9–25·4	87·8–95·6	0·08–0·16

### Membrane filtration method

A simple MF protocol adapted recently for the lake Neusiedler See (Schauer *et al*. 2012, 2015) was used. For all samples, four sample volumes (100, 10, 1 and 0·1 ml; the two lower volumes filled up to 10 ml with 1x PBS for an even distribution on the filter) were directly filtered through 0·45‐*μ*m pore size nitrocellulose filters (47 mm diameter), transferred to TCBS (Merck, Vienna, Austria) agar plates, and incubated for 18–24 h at 37 ± 1°C. In preliminary investigations TCBS agar from two companies (Merck, Vienna, Austria, product nr: 1·10263; Sigma‐Aldrich, Vienna, Austria, product nr: 86348) was tested for several sites of lake Neusiedler See. It became clear that the product from Sigma‐Aldrich is less selective (likely due to differences in the chemical composition) and when low numbers of *V. cholerae* are present (beginning and end of the warm season) they are often overgrown by competitive species with a similar colony appearance, mainly *Aeromonas* sp. and *Exiguobacterium* sp. (Fig. S1). During summer, on the other hand, the Sigma‐Aldrich agar resulted in slightly higher concentrations (average difference 0·3 log) than the Merck agar (Mann–Whitney *U*‐test, *P* < 0·001), most probably due to the fact that the higher selectivity of the Merck agar prevented colony growth of stressed *V. cholerae*. When starting with the investigations in 2016 we used parallel plating on both agars for representative samples and again observed overgrowth of the Sigma‐Aldrich agar with *Aeromonas* sp. and *Exiguobacterium* sp. masking the presence of *V. cholerae*. We, therefore, decided to use Merck agar throughout the study.

Typical *V. cholerae* colonies on TCBS (yellow, diameter 2–3 mm, shiny, sharp‐edged, slightly raised) were counted and 10 representative colonies per site were streaked onto nutrient agar plates without added NaCl (3% beef extract, 5% peptone, 15% agar) and incubated overnight at 37°C. As *V. cholerae* is able to grow in the absence of NaCl (Baron *et al*. 2007; ISO 2007; CDC 2014), colonies growing on agar without NaCl were considered presumptive *V. cholerae*. Presumptive isolates were confirmed by both MALDI‐TOF (MALDI Biotyper; Bruker Daltonik GmbH, Bremen, Germany) and a species‐specific multiplex PCR targeting ompW for species identification (Nandi *et al*. 2000; Baron *et al*. 2007), as well as tcp, ctxA (cholera toxin), wbe (O1 antigen) and wbf (O139 antigen) (Bliem 2009) (Table S2).

### Standard MPN method

Standard determination and identification of *V. cholerae* was performed via a triplicate MPN method in accordance with the British national standard method (Public Health England 2005). Triplicate 1‐ml samples were serially diluted (1 : 1, 1 : 10 and 1 : 100) in APW and the triplicate 1‐ml samples of each dilution were inoculated into 9 ml APW enrichment broth. Each tube was incubated for 18–24 h at 30 ± 1°C and observed for the presence or absence of growth. Surface aliquots from the microaerophilic pellicle layer (Huq *et al*. 2012) of each tube were streaked out on TCBS agar plates (Oxoid, Wesel, Germany, CM0333). After 24 h at 37 ± 1°C presumptive *V. cholerae* colonies from all positive MPN tubes were transferred to nutrient agar without NaCl (Columbia agar+5% sheep blood; BioMérieux, Marcy l'Etoile, France). When growth was observed, the isolates were identified with specific tests: oxidase, API 20 E, O1/O139 serum agglutination test and MALDI‐TOF (Bruker Daltonik). Final MPN results were obtained from using a three‐tube MPN table according to the methods recommended by the US Food and Drug Administration (FDA 2010).

### Direct plating

For DP, 1 ml of sample was spread over the TCBS agar plate (Oxoid, CM0333) with a sterile Drygalski spatula under circular motions. After incubation at 37 ± 1°C for 18–24 h the typical colonies were counted and three representative colonies were transferred to nutrient agar without NaCl and analysed and identified as described above.

### Sample limit of detection and confidence intervals

The sample limit of detection (SLOD) of the threefold MPN approach used (sample volume 1 ml, three dilutions) was 30 MPN per 100 ml, with the simple DP approach (sample volume 1 ml, one replicate) it was 300 CFU per 100 ml. With the chosen MF approach (triplicates, maximal sample volume 100 ml, four dilutions) the SLOD was 3 CFU per 100 ml. The value that was defined as SLOD was the number that is—for the respective sample volume—statistically significant from zero with a probability of >95% (ISO 2000). Based on the assumed random distribution of bacteria in water, a colony count of ≥3 is statistically significant from zero. Thus, the SLOD is 3 CFU per 100 ml for the MF approach and 3 CFU per 100 ml for the DP approach, corresponding to 300 CFU per 100 ml. If, however, in the investigated volume one or two colony‐forming units are randomly present, a positive detection of *V. cholerae* below the theoretical SLOD can occur. The 95% confidence intervals of the MPN approach were simply taken from the MPN table published by the FDA (FDA 2010). The MF 95% confidence intervals were calculated as 1·96× standard deviation (Gosling 1995). For the DP approach no confidence intervals can be given, as only a single replicate was analysed.

### Statistical analysis

All statistical analysis was performed with ibm spss 23. For correlation analysis, Spearman‐rank correlation coefficients (rho) were calculated. Kruskal–Wallis H‐tests were used for comparing results obtained by the three different methods. For this comparison only those samples were used where all three methods gave a positive result. For prediction of *V. cholerae* abundance from environmental parameters, multiple linear stepwise regression models were calculated with the results from the MF method. Results were considered significant at a probability level <0·05.

## Results

**Figure 1:**
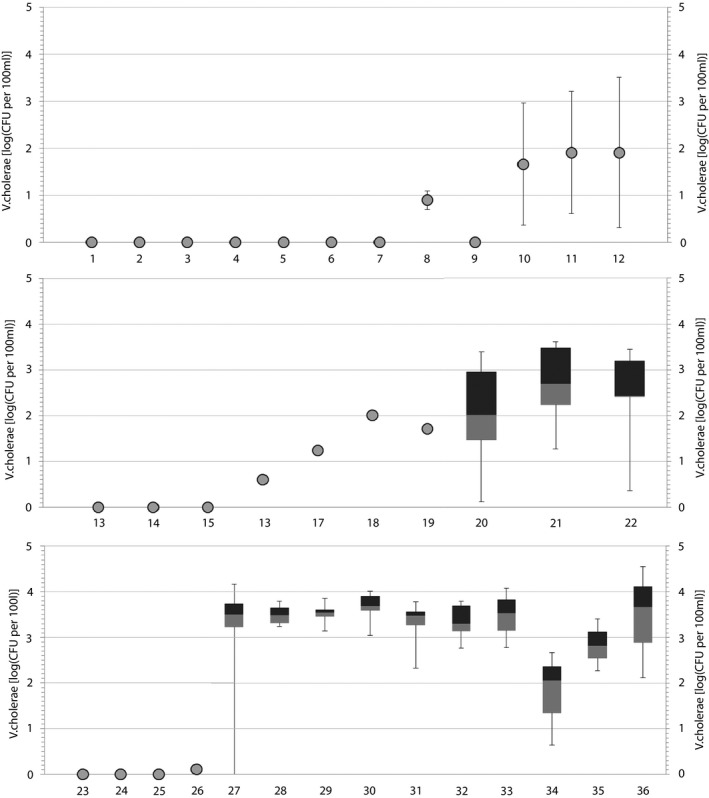
Abundance of culturable *Vibrio cholerae* nonO1/nonO139 at 36 Eastern Austrian bathing sites, determined with the membrane filtration protocol. For bathing sites sampled at one single day, the average value of triplicate samples (grey circles) ±1 standard deviation is depicted. For all bathing sites sampled multiple times, data are depicted in box‐plots. The box‐plots show median, 25 and 75% percentiles as well as minimum and maximum values of three (sites 34–36), five (sites 20–22) and six seasonal triplicate measurements (sites 27–33).

Culturable *V. cholerae* nonO1/nonO139 were present at 22 of the 36 investigated bathing sites (Fig. 1). At 14 sites, no *V. cholerae* could be detected with the MF method; at these sites *V. cholerae* were also neither detected with the MPN standard approach nor with the DP approach. Concentrations ranged from below the theoretical SLOD of 3 CFU per 100 ml (site 26; 1 CFU per 100 ml) to 39 000 CFU per 100 ml (site 36, August value).

### Comparison of detection methods

**Figure 2:**
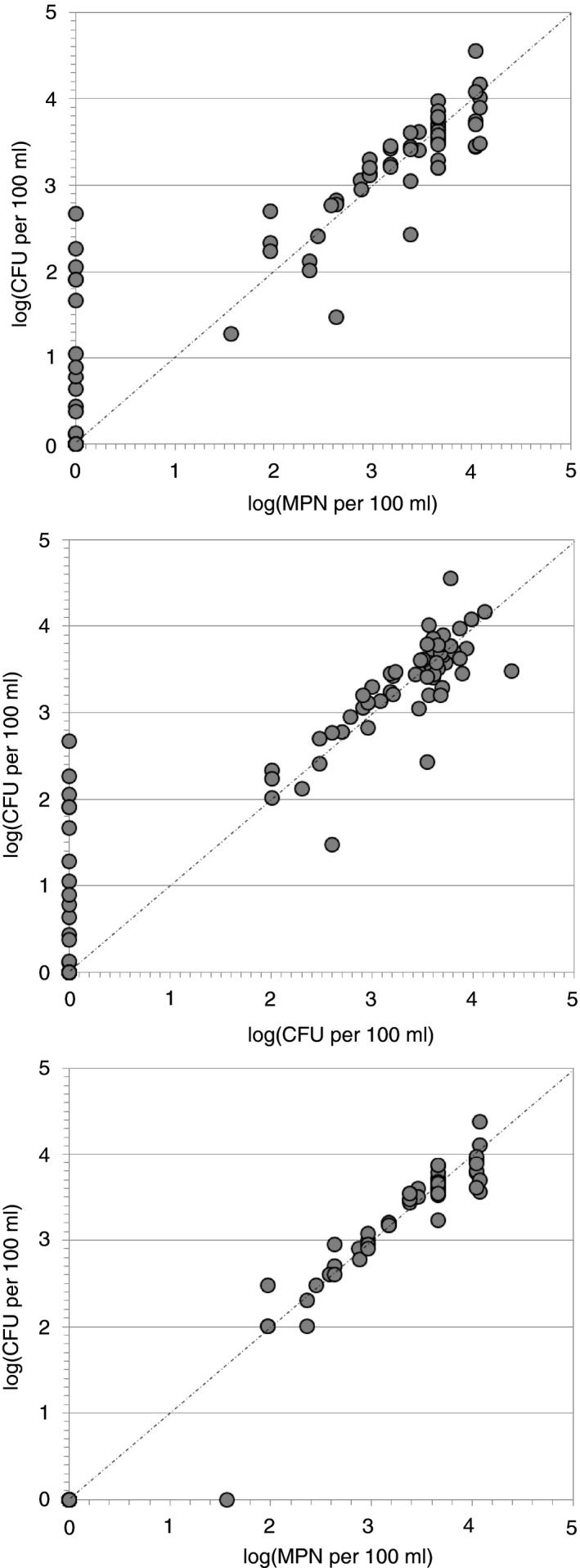
Scatter plots of the correlations between the three applied methods for enumeration of *Vibrio cholerae*. (a) MPN (*x*‐axis) *vs* MF (*y*‐axis); (b) DP (*x*‐axis) *vs* MF (*y*‐axis); (c) MPN (*x*‐axis) *vs* DP (*y*‐axis); for the MPN method, each value >11 000 MPN per 100 ml was transformed to 12 000 MPN per 100 ml. The dashed line depicts the 1 : 1 line.

In general, the three cultivation methods yielded comparable results, when the different detection limits were taken into consideration (Fig.**** 2). There was no significant difference between the results obtained by the three different methods (Kruskal–Wallis H‐test, *P* > 0·1). Highly significant correlations were observed between the MF results and the results obtained with the MPN (Spearman rho = 0·90; *P* < 0·001) and DP assay (rho = 0·88; *P* < 0·001). However, *V. cholerae* were not detected with MPN or DP in 15 and 16 samples, respectively, despite a positive finding with MF. An excellent correlation was observed between the MPN and the DP assay (rho = 0·97; *P* < 0·001) and a 95% accordance of culture‐negative samples (Fig. 2). With the applied settings (three replicates, 1 ml per enrichment vial, three dilutions) the MPN approach used delivered imprecise results at high *V. cholerae* concentrations: all concentrations ≥4600 MPN per 100 ml had statistical MPN values of either 4600, 11 000 or >11 000 MPN per 100 ml. For operational reasons to enable statistical comparison between methods the four values >11 000 MPN per 100 ml were defined to be 12 000 MPN per 100 ml. Over the whole range of concentrations, the 95% confidence intervals of the MF method were much smaller than for the MPN method (Table S1). For values above 30 CFU per 100 ml, the median upper 95% confidence interval of the MF method was 136% of the average of triplicate measurements (range 102–262%), while the median upper confidence interval of the MPN method was 401% of the calculated MPN (range: 267–500%). No confidence interval could be calculated for the DP approach, as only one replicate was analysed.

All presumptive *V. cholerae* isolates from the MF approach that grew on nutrient agar without NaCl were identified both via MALDI–TOF and PCR analysis. In total, 802 isolates were analysed, with 800 matches (99·75% coincidence), among them 644 *V. cholerae* positive and 156 *V. cholerae* negative matches, identified as either *Aeromonas* sp. or *Exiguobacterium* sp. by MALDI‐TOF. *Vibrio cholerae* concentrations were corrected for false‐positive colonies. In two cases a positive result was obtained with MALDI‐TOF, and a negative with PCR. None of the *V. cholerae* isolates did possess ctxA, tcp, wbe or wbf.

### Influence of environmental factors on *V. cholerae* abundance

**Figure 3:**
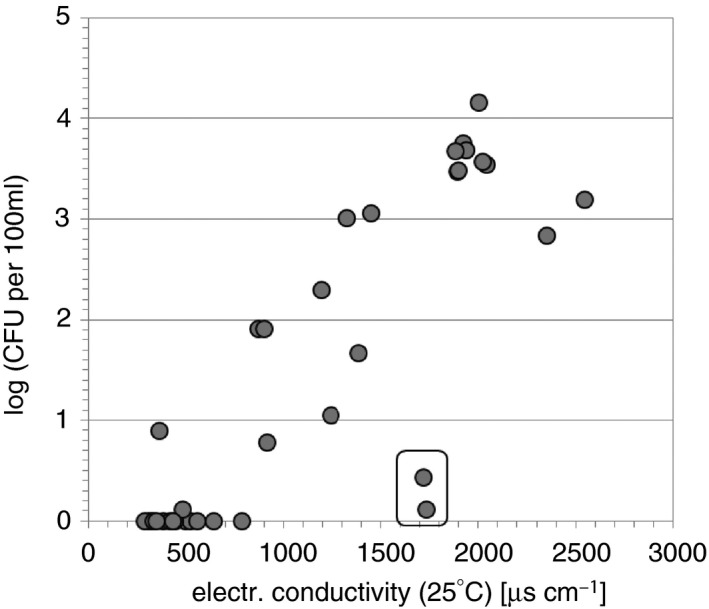
Scatter plot between electrical conductivity and *Vibrio cholerae* abundance determined with the membrane filtration method. The box indicates the two environments with low *V. cholerae* abundance despite high conductivity values.

To find out the determining factors driving culturable *V. cholerae* abundance, simple correlation analysis was performed in a first step. When all individual measurements were considered (*n* = 89), all measured environmental water parameters were significantly correlated with *V. cholerae* abundance, determined with all three methods (Table 2a). Electrical conductivity and pH values correlated positively, water temperature, Secchi depth and oxygen content correlated negatively with *V. cholerae* abundances. In order to prevent overweighting of sites with multiple measurements, correlation was also performed at the basis of individual bathing sites (*n* = 36; average values were used for bathing sites with multiple measurements over the season), and a similar pattern was observed (Table 2b). The surprising negative correlation with temperature for the data set of the individual bathing sites was only illusive and caused by the specific sampling design: the 23 bathing sites measured once at high temperature conditions (24–27°C; see above) had to a large extent low *V. cholerae* abundances or were negative, while the bathing sites with high *V. cholerae* abundances were sampled more often covering also periods with relatively lower temperature (<22°C). Similarly, the negative correlation with Secchi depth resulted from repeated sampling of various extremely shallow, turbid environments (water depth below 1·5 m, Secchi depth varying between 8 and 30 cm) with high *V. cholerae* abundances, while many environments with higher water transparency (i.e. Secchi depth) exhibited low *V. cholerae* abundances. Those turbid environments usually also prevent intensive algal growth and oxygen production due to light limitation, leading to an illusive negative correlation of *V. cholerae* abundances with oxygen. Both, pH and electrical conductivity were positively correlated with *V. cholerae* abundances with conductivity showing the highest correlation coefficients (rho = 0·862 when pooled data for each individual bathing site was considered; see Table 2b; Fig. 3). From Fig. 3 it became evident that substantial *V. cholerae* concentrations above 2 log_10_ per 100 ml occurred only at bathing sites with an electrical conductivity value >1200 *μ*S cm^−1^ (approx. 0·6 ppt salinity). Nevertheless, there were also a few environments with <1000 *μ*S cm^−1^, where *V. cholerae* was detected at low numbers (<2 log_10_ per 100 ml). Even at 360 cm^−1^, 6·9 ± 2·3 CFU per 100 ml were observed at one sampling site. On the other hand, there were two environments with a relatively high conductivity (>1700 *μ*S cm^−1^; approx. 0·86 ppt salinity), where also only low numbers of *V. cholerae* were observed (marked with a box in Fig. 3).

**Table 2:** Spearman rank correlation analysis of *Vibrio cholerae* abundances determined with the three different methods and the measured ecological variables. (a) All single measurements were considered (*n* = 89); (b) single or average values (from multiple seasonal sampling) for each bathing site were considered (*n* = 36)

(a) All measurements (*n* = 89)	O_2_	pH value	Water temp	Secchi depth	Electr. cond
*V. cholerae* MF					
rho	−0·590	0·458	−0·198	−0·604	0·647
*P*‐value	0·000	0·000	0·063	0·000	0·000
*V. cholerae* MPN					
rho	−0·559	0·500	−0·177	−0·645	0·614
*P*‐value	0·000	0·000	0·098	0·000	0·000
*V. cholerae* DP					
rho	−0·528	0·521	−0·185	−0·640	0·581
*P*‐value	0·000	0·000	0·083	0·000	0·000

(b) All bathing sites (*n* = 36)	O_2_	pH value	Water temp	Secchi depth	Electr. cond
*V. cholerae* MF					
rho	−0·535	0·522	−0·400	−0·547	0·862
*P*‐value	0·001	0·001	0·016	0·001	0·000
*V. cholerae* MPN					
rho	−0·629	0·467	−0·561	−0·619	0·771
*P*‐value	0·000	0·004	0·000	0·000	0·000
*V. cholerae* DP					
rho	−0·618	0·441	−0·567	−0·612	0·751
* P*‐value	0·000	0·007	0·000	0·000	0·000

Considering all measured environmental data, we tried to establish a preliminary prediction model for *V. cholerae* abundances in Eastern Austrian bathing sites, based on multiple stepwise linear regression. Due to the illusive relationships mentioned above, only electrical conductivity remained as a significant predictor of *V. cholerae* abundance. Electrical conductivity explained 73% of the variability in the observed *V. cholerae* abundance in the investigated environments, covering a range of approximately 0·14–1·4 ppt salinity. Obviously, other parameters may also play a significant role controlling the *V. cholerae* nonO1/nonO139 abundance in some of the bathing waters in Eastern Austria. Such factors could be the pH for which a significant correlation was observed, and factors that were not determined in the present study like the quantity and quality of organic matter, the presence of specific algae or zooplankton or the specific composition of the salinity.

## Discussion

### Culturable *V. cholerae* nonO1/nonO139 are widely prevalent in Eastern Austrian bathing waters

From 36 investigated Eastern Austrian bathing sites, culturable *V. cholerae* were found at 22 sites. Abundances ranged from 1 to 39 000 CFU per 100 ml. This maximum value was of comparable magnitude as the maximum observed in the Austrian Lake Neusiedler See during 2011/2012 (30 000 CFU per 100 ml; (Schauer *et al*. 2015)) but about one order of magnitude lower than the maximum concentrations (3 × 10^6^–6 × 10^6^ cells per CFU per litre) observed so far for other (estuarine and coastal) ecosystems (Jiang and Fu 2001; Heidelberg *et al*. 2002; Neogi *et al*. 2012). Up to now, it is not clear how *V. cholerae* nonO1/nonO139 abundance relates to disease risk. In Austria, notified *V. cholerae* infections (mild gastrointestinal infections, wound infections, ear infections, necrotizing fasciitis) have only been associated with bathing waters with rather high *V. cholerae* concentrations, the Lake Neusiedler See (Huhulescu *et al*. 2007) and two slightly saline small ponds, where maximum concentrations of >11 000 MPN per 100 ml were found in 2015 (Hirk *et al*. 2016). For the Baltic Sea—as the most prominent example—where dramatically increasing numbers of *V. cholerae* infections have been reported in recent years (Baker‐Austin *et al*. 2013, 2016b), no quantitative data on *V. cholerae* concentrations are available. In addition, no information is available on the dose–response relationships of *V. cholerae* nonO1/nonO139 in general and of different *V. cholerae* serogroups/strains specifically. It is plausible to assume that the infection dose for a gastrointestinal infection is significantly higher than for a wound infection due to the stomach acid barrier. Dose–response relationships only exist for cholera and lie in the range from 10^5^ to 10^6^ cells (Sack *et al*. 1998; Cohen *et al*. 1999). For wound infections only a few cells may be sufficient, especially in susceptible individuals. Moreover, severe differences between strains in their capabilities to cause infection may exist. Schirmeister *et al*. (2014), for example, showed that strains from diarrhoeal patients possessed the type III secretion system and/or the multifunctional autoprocessing repeats‐in‐toxin (MARTX), which were not found in the strains from ear or wound infections. Different strains with different virulence potential may be present simultaneously in bathing waters, as it is known that *V. cholerae* intraspecies diversity is high (Keymer and Boehm 2011; Pretzer *et al*. 2017). Whole genome sequencing used for typing purposes (core genome multilocus sequence typing; cgMLST) and identification of virulence factors within the genomes are currently underway to assess the virulence potential of the diverse strains isolated in this study. So, due to the big lack of knowledge and the complex biological situation a risk assessment simply based on *V. cholerae* concentrations in bathing waters is currently not possible. It has to be added that at unfavourable environmental conditions (e.g., low temperature) *V. cholerae* may switch to the viable but nonculturable (VBNC) state (Wu *et al*. 2016) where they cannot be detected with culture‐based methods but where they may retain their virulence (Colwell *et al*. 1985). The true prevalence and abundance of *V. cholerae* can thus be underestimated by using only cultivation‐based methods (Schauer *et al*. 2015; Bliem *et al*. 2018).

### Conductivity is the most important factor explaining *V. cholerae* abundance in Austrian bathing waters at warm water temperatures

In a comprehensive review Takemura *et al*. (2014) have elaborated that *V. cholerae* abundance in aquatic environments worldwide was best explained by temperature, the presence of other organisms and salinity. Due to the fact that most samples were taken during the warm season, no correlation with temperature was observed in our study. When water temperatures are warm enough for *V. cholerae* growth, conductivity (as measure of salinity) seems to be the most important factor explaining the prevalence and abundance of *V. cholerae* in Eastern Austrian bathing waters. A highly significant correlation was observed between *V. cholerae* abundance and electrical conductivity both when all single measurements were considered and when pooled data for each environment was used. With one exception, values above 1 CFU per 100 ml only occurred at conductivities above 900 *μ*S cm^−1^, high *V. cholerae* concentrations (>10^2^ CFU per 100 ml) were only observed above 1200 *μ*S cm^−1^. It has been reported that *V. cholerae* prefers brackish water conditions with salinities between approx. 1–14 ppt, corresponding to about 2000–23 000 *μ*S cm^−1^ at 25°C (Louis *et al*. 2003; Takemura *et al*. 2014), but effective multiplication in freshwater has also been demonstrated (Vital *et al*. 2007). The measured electrical conductivity values of 1000 *μ*S cm^−1^ correspond to approximately 0·5 ppt salinity and are thus below or at the lower end of the reported salinity optimum. Whether higher conductivities above the maximum value observed in this study (2656 *μ*S cm^−1^, corresponding to approx. 1·4 ppt) would promote higher *V. cholerae* abundances cannot be predicted because no such bathing sites have been identified in Eastern Austria so far. Surprisingly, there were also two environments with relatively high conductivity values (~1700 *μ*S cm^−1^, approx. 0·86 ppt) that showed *V. cholerae* concentrations below the SLOD of 3 CFU per 100 ml. On the other hand, there was one environment with a conductivity value of only 360 *μ*S cm^−1^ that had *V. cholerae* concentrations of ~7 CFU per 100 ml. Obviously, other ecological factors may also be of crucial importance. From the parameters considered in this study only pH—next to conductivity—showed a positive correlation, all other parameters (temperature, oxygen, water transparency) exhibited illusive or indirect negative correlations to *V. cholerae* abundance that was caused by the specific sampling design (see Results section). *Vibrio cholerae* preferentially grow in environments with alkaline pH (Huq *et al*. 1984) and such conditions (8·6 ± 0·2) are even used for selective enrichment of Vibrios in the laboratory (CDC 2014). The pH values observed in the studied environments ranged from 7·96 to 9·18 with the majority falling into the range from 8·2 to 9·0, thus providing rather optimal conditions. Also, the pH of the two bathing sites that had low *V. cholerae* abundance despite high conductivity fell within this range.

It is well known that temperature positively influences *V. cholerae* abundance and growth (Huq *et al*. 1984; Takemura *et al*. 2014; Schauer *et al*. 2015), while *V. cholerae* as a facultative anaerobic bacterium is adapted to a wide range of oxygen conditions (CDC 2014). So, due to the specific sampling design these two parameters were not meaningful for explaining the distribution of *V. cholerae* in Eastern Austrian bathing waters. Other parameters that were not determined in the present study like the quantity and quality of organic matter (Kirschner *et al*. 2008), the presence of phytoplankton (Islam *et al*. 1999; Eiler *et al*. 2007) or zooplankton (Tamplin *et al*. 1990; Kirschner *et al*. 2011), or the specific ion composition (Schauer *et al*. 2015) will have to be considered in future investigations.

In an attempt to derive an empirical formula to predict *V. cholerae* concentrations as a function of the measured environmental variables (Sterk *et al*. 2015), multiple stepwise regression analysis was performed. In this analysis, only conductivity remained as a significant predictor, explaining 73% of the variance of *V. cholerae* abundance. More detailed monitoring with a higher seasonal resolution is necessary to set‐up robust prediction models for the presence and abundance of *V. cholerae* nonO1/nonO139 in Austrian bathing waters.

### Membrane filtration and direct plating are superior to the standard MPN approach

In general, the three applied methods to enumerate *V. cholerae* in water samples yielded coincident results. No significant differences and highly significant correlations between the different methods were observed with the highest accordance between the MPN and the DP approach. This was caused by the fact that these two methods were applied for the same sample while the MF method was applied for triplicate additional samples. It was interesting to see that the MPN assay that includes an enrichment step did not lead to higher values than the two methods without enrichment. It has often been assumed that effective detection of *V. cholerae* (especially toxigenic strains) requires an enrichment step. The results here suggest that may not be the case universally. Differences were observed between the methods concerning the detection limit and the confidence intervals. With the chosen sample volumes (0·1–100 ml for MF, 3 × 1 ml and two 10‐fold serial dilutions for MPN and 1 ml for DP), the MF approach had a much lower SLOD (3 CFU per 100 ml) than the MPN (30 MPN per 100 ml) or the DP approach (300 CFU per 100 ml). Therefore, many samples were negative with the MPN (*n* = 15) and DP approach (*n* = 16), while the MF method delivered positive results. In this context it has to be mentioned that the choice of the TCBS agar can be of critical influence. Using an agar with lower selectivity (e.g. Sigma‐Aldrich, product nr: 86348) may lead to overgrowth of the agar plate with competitive bacteria (mainly *Aeromonas* sp., *Exiguobacterium* sp.) masking the presence of *V. cholerae* (Fig. S1). Using an agar with higher selectivity (Merck product nr: 1.10263) leads to a better detection of *V. cholerae* at low abundances but also to a slight underestimation of colony‐forming units at higher abundances (Fig. S1). Increasing the sample volume to 10 ml and the number of replicates to five for the MPN approach would solve the problem with the high detection limit, but would significantly increase the effort and costs of the analysis hampering the simultaneous analysis of a large number of samples. For the DP approach, the sample volume cannot be increased as the maximum volume that can be spread over the agar surface is 1 ml.

Nitrocellulose filters with a nominal pore size of 0·45 *μ*m were used in this study. The use of such filters is the gold‐standard for the determination of many water bacteria (Clesceri *et al*. 1998). These filters provide a multilayered web of fibres in which also smaller particles are entrapped. Huq *et al*. (2012) recommended the use of 0·2 *μ*m polycarbonate filters against nitrocellulose filters because the bacteria can then be better removed by vortexing and are not trapped within the membranes, necessary for transfer to a subsequent enrichment broth. However, in our study, we directly transferred the filters on a TCBS agar plate without the necessity of removing the cells. In contrast to polycarbonate filters, nitrocellulose filters on the surface of nutrient agar plates allow sufficient nutrients from the plate to diffuse through the filters (along the moist cellulose fibres) and support bacterial growth. Certainly we cannot exclude the loss of small starved *V. cholerae* cells, but we think that they represent a minor fraction of the total population. There were no significantly lower numbers obtained by the MF method in comparison to DP or MPN, where samples are directly inoculated in/on the growth medium (Kruskal–Wallis H‐test, *P* > 0·1).

Confidence intervals of the triplicate MF approach (102–262% of the measured value) were much lower than the ones of the three‐tube MPN approach (267–500%), which partly gives only rough estimations of the abundance of culturable *V. cholerae*. Using a five‐tube MPN approach would lower the confidence intervals to approximately 230–300% but would still be higher than the intervals observed for the MF method. No confidence intervals could be calculated for the DP approach used, as only a single sample was analysed, but it can be assumed that the variability is similar to the MF approach, where bacteria are spread over the whole surface of a filter.

We, therefore, recommend the chosen MF approach as a reliable, sensitive and relatively easy method for the enumeration of *V. cholerae* in water samples, covering a wide range of ecological conditions, from freshwater to brackish water, from river water to lake/pond water and from transparent to moderately turbid waters, where formation of colonies is not precluded by clogged filter membranes. If high numbers of *V. cholerae* are expected, the analysis of triplicate samples per site can be omitted and an average coefficient of variance of 36% for a single analysis can be assumed, as observed for the actual data set. If low numbers around the SLOD are expected, triplicate analysis make sense as it increases the probability of a positive finding. To further lower the SLOD of the MF method, higher sample volumes could be analysed, provided that they are still filterable through the 0·45‐*μ*m pore size filter used and that overgrowth of other microbiota do not prohibit the identification of *V. cholerae* colonies. We further suggest using a more selective TCBS medium to prevent such overgrowth as we often achieved negative results with a less selective TCBS agar. Due to the fact that multiplex PCR and MALDI‐TOF yielded nearly identical results, identification can be equivalently performed with both methods. MALDI‐TOF is quicker and cheaper than multiplex PCR, on the other hand, the multiplex PCR used delivers information on the presence of virulence genes (ctx, tcp) and whether the strains belong to the serogroups O1, O139 or are nonO1/nonO139 strains. The simplest procedure was the DP method that can be recommended as a rapid and easy screening tool when concentrations above an SLOD of 300 CFU per 100 ml are expected. In contrast, the used standard three‐tube MPN method is comparably imprecise and elaborate and did not result in statistically significant higher numbers than the other methods without pre‐enrichment to repair injured or resuscitate VBNC cells. It can, therefore, not be recommended for the monitoring of *V. cholerae* presence and abundance in bathing water. Analysing more replicates and higher volumes (e.g. a five‐tube MPN with 10 ml each) would lead to more precise results but would exorbitantly increase costs and efforts.

### Practical implications of the study

In order to use noncholera vibrios as barometers of climate change (Baker‐Austin *et al*. 2016a) and to prove that the emerging noncholera vibrio infections are indeed related to climate change‐induced temperature increase in marine and inland waters, reliable quantification methods for environmental samples are necessary. Such quantification methods can be cultivation‐independent methods like, for example, fluorescence *in situ* hybridization combined with cytometry (Schauer *et al*. 2012) and qPCR (Bliem *et al*. 2015) or traditionally culture‐based. In the present study, we present an environmental survey of *V. cholerae* nonO1/nonO139 enumeration at 36 bathing sites with three different culture‐based methods, in which a rather simple MF protocol yielded reliable and sensitive results with a better performance than the standard MPN approach. For further investigations, this approach should be tested in interlaboratory trials in order to develop an easy, sensitive and reliable standard procedure for the quantification of *V. cholerae* in water samples. The DP method—as a rapid and easy screening tool—may be a good alternative for samples with presumptive high *V. cholerae* nonO1/nonO139 concentrations.

With the MF method used we could show that *V. cholerae* are widely present in bathing waters in Eastern Austria, with concentrations ranging from 1 CFU to 39 000 CFU per 100 ml. Thus, these pathogens are more widespread than previously thought and could pose a health risk to susceptible persons at least at those bathing waters where high concentrations were found. For the obtained data set, conductivity was the factor most strongly correlated with *V. cholerae* presence in the different bathing waters at warm water temperatures, but other factors may play an important role as well. More detailed monitoring, based on better seasonal resolution and including more ecological variables, is necessary to set‐up prediction models for the presence and abundance of *V. cholerae* nonO1/nonO139 in Austrian bathing waters. Finally, for a potential quantitative microbial risk assessment, information gaps have to be filled. These include gaps concerning (i) hazard identification, for example, the presence of strains with different virulence potential and the composition of its virulence factors, (ii) exposure assessment, for example, how many *V. cholerae* cells a swimmer has contact to during water exposure, depending on oral uptake, body shape, distribution of cells in the water, etc. and (iii) dose–response relationships, for example, how many *V. cholerae* nonO1/nonO139 cells are necessary to cause gastrointestinal or wound infections.

## Conflict of Interest

The authors declare no conflict of interest.

## Supporting information


**Figure S1** Comparison of culturable *Vibrio cholerae* abundances at four sites of the Lake Neusiedler See in 2014, determined with the membrane filtration method using TCBS agar from two different companies: Merck (product nr: 1.10263) and Sigma‐Aldrich (product nr: 86348).
**Table S1** *Vibrio cholerae* concentrations and 95% confidence intervals (CI) at the 36 investigated Eastern Austrian bathing sites determined with the membrane filtration method (MF), the most probable number method (MPN) and the direct plating method (DP).
**Table S2** Primers and PCR conditions used for the multiplex PCR for identification of presumptive *Vibrio cholerae* isolates.
