# Supplementary material for: Application of three different methods to determine the prevalence, the abundance and the environmental drivers of culturable Vibrio cholerae in fresh and brackish bathing waters
Source: J Appl Microbiol. 2018 Aug 13;125(4):1186–98. doi: 10.1111/jam.13940 (PMC6175421; doi:10.1111/jam.13940)
Supplement: Supplementary file 1 — Figure S1 Comparison of culturable Vibrio cholerae abundances at four sites of the Lake Neusiedler See in 2014, determined with the membrane filtration method using TCBS agar from two different companies: Merck (product nr: 1.10263) and Sigma‐Aldrich (product nr: 86348). Table S1 Vibrio cholerae concentrations and 95% confidence intervals (CI) at the 36 investigated Eastern Austrian bathing sites determined with the membrane filtration method (MF), the most probable number method (MPN) and the direct plating method (DP). Table S2 Primers and PCR conditions used for the multiplex PCR for identification of presumptive Vibrio cholerae isolates. [file JAM-125-1186-s001.docx]

**Supplemental Information**

Kirschner AKT, Hirk S, Jakwerth S, Rehak S, Farnleitner AH, Huhulescu S, Indra A: Application of three different methods for studying the prevalence and abundance of culturable *Vibrio cholerae* in fresh and brackish bathing waters.

**Figure S1:** Comparison of culturable *V. cholerae* abundances at four sites of the Lake Neusiedler See in 2014, determined with the membrane filtration method using TCBS agar from two different companies: Merck (product nr: 1.10263) and Sigma-Aldrich (product nr: 86348).


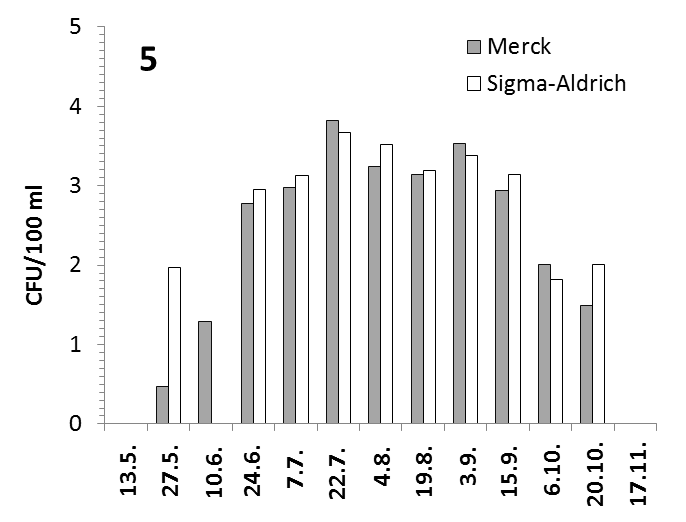

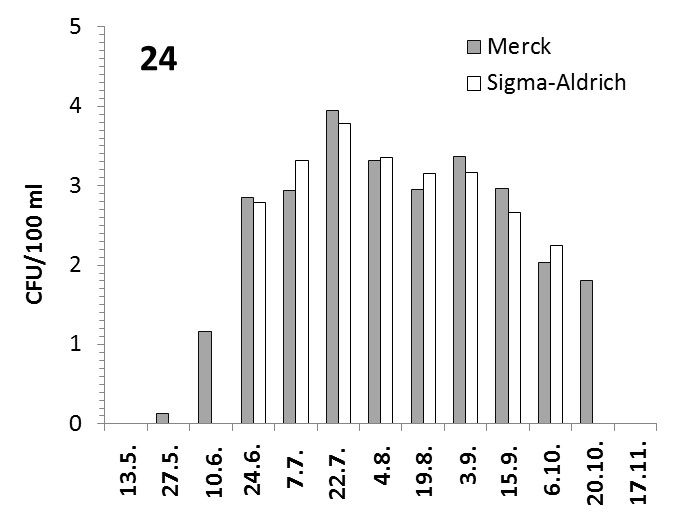

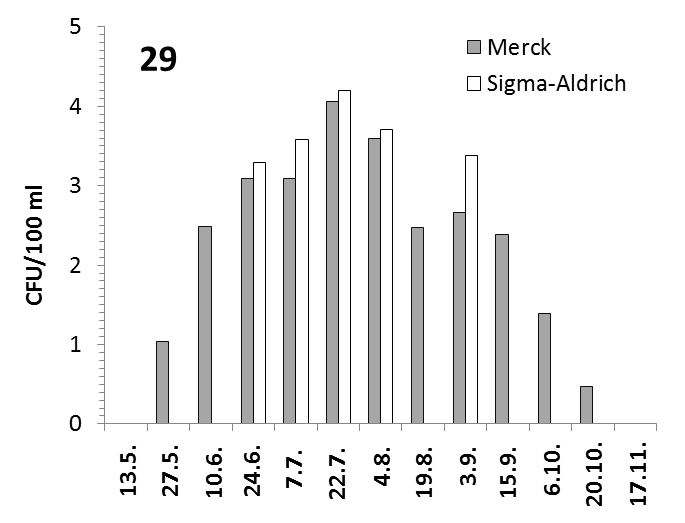

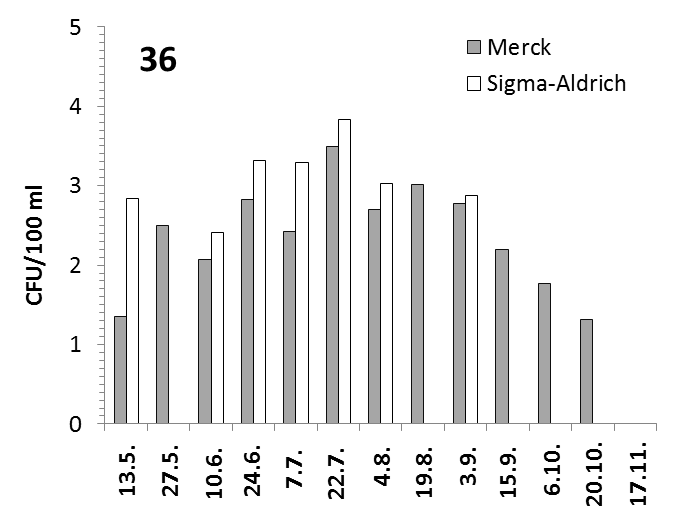


**Table S1:** *V. cholerae* concentrations and 95% confidence intervals (C.I.) at the 36 investigated Eastern Austrian bathing sites determined with the membrane filtration method (MF), the most-probable number method (MPN) and the direct plating method (DP). Values in brackets indicate values below the defined sample limit of detection (SLOD).

**Table S2:** Primers and PCR conditions used for the multiplex PCR for identification of presumptive *V. cholerae* isolates.

| Target | Nucleotide sequence | bp | Reference |
| --- | --- | --- | --- |
| ompW (F)  ompW (R) | CAC CAA GAA GGT GAC TTT ATT GTG  GGT TTG TCG AAT TAG CTT CAC C | 304 | Nandi et al. 2000 |
| ctxAB (F)  ctxAB (R) | GCC GGG TTG TGG GAA TGC TCC AAG  GCC ATA CTA ATT GCG GCA ATC GCA TG | 536 | Goel et al. 2007 |
| wbe O1 (F)  wbe O1 (R) | TCT ATG TGC TGC GAT TGG TG  CCC CGA AAA CCT AAT GTG AG | 638 | Goel et al. 2007 |
| wbf O139 (F)  wbf O139 (R) | AGC CTC TTT ATT ACG GGT GG  GTC AAA CCC GAT CGT AAA GG | 449 | Hoshino et al. 1998 |
| tcp (F)  tcp (R) | CGT TGG CGG TCA GTC TTG  CGG GCT TTC TTC TTG TTC G | 805 | Goel et al. 2007 |

All isolates were boiled for 10 min and after centrifugation at 13.000 rcf (10 min, 4°C) the supernatant was used as a DNA template for Multiplex PCR. Multiplex PCR was performed with a T3, Biometra® gradient thermocycler (Biometra®, Göttingen, Germany).

The following reagents were used for multiplex PCR in a 200 µl PCR tube containing a reaction volume of 25 µl: 2.5 µL of a 10 x reaction buffer B (Mg2+ free) [0.8 M Tris-HCl, 0.2 M (NH_4_)_2_SO_4_, 0.2% w/v Tween-20, Solis BioDyne, Tartu, Estonia], 2 µL of 25 mM MgCl_2_ (Solis BioDyne, Tartu, Estonia), 0.5 µL of 10mM dNTP mix (Promega, Mannheim, Germany), 1.2 µl each of the forward and reverse primer of ompW and tcp (10 pmol µL^-1^), 1 µl each of the O1 wbe primer pair (10 pmol µL^-1^) and O139 wbf primer pair (10 pmol µL^-1^) and 0.9 µL for the ctxA primer pair (10 pmol µL^-1^), 0.4 µL of hot FIREPol® DNA polymerase (Solis BioDyne, Tartu, Estonia) and 5 µL of DNA template and Mili-Q water to a final volume of 25 µL.

The thermocycler conditions used were 94°C for 15 min, followed by 30 cycles consisting of 94°C for 1 min, 59°C for 1min and 72°C for 2 min and a final extension step at 72°C for 10 min at the end of 30 cycles, followed by maintenance at 4°C.

Multiplex PCR products were separated by agarose gel electrophoresis (2%) for 70 min with 90 V in 1 x TAE buffer 40 mM Tris, 1 mM EDTA, pH 8), stained in 1 µg ml^-1^ ethidium bromide solution and visualized under UV light (GelDoc 2000 System; Biorad, Hercules, CA).

**References**

Goel AK, Ponmariappan S, Kamboj DV, Singh L (2007) Single Multiplex Polymerase Chain Reaction for Environmental Surveillance of Toxigenic-Pathogenic O1 and Non-O1 *Vibrio cholerae*. Folia Microbiol 52, 81-85

Hoshino K, Yamasaki S, Mukhopadhay AK, Chakraborty S, Basu A, Bhattacharya SK, Nair GB, Shimada T & Takeda Y (1998) Development and evaluation of a multiplex PCR assay for rapid detection of Toxigenic Vibrio cholerae O1 and O139. FEMS Immunol Med Mic 20: 201–207

Nandi B, Nandy R, Mukhophyay S, Nair G, Shimada T, Ghose A (2000) Rapid method for species-specific identification of Vibrio cholerae using primers targeted to the gene of outer membrane protein OmpW. J Clin Microbiol 38: 4145-4151
